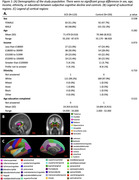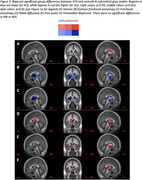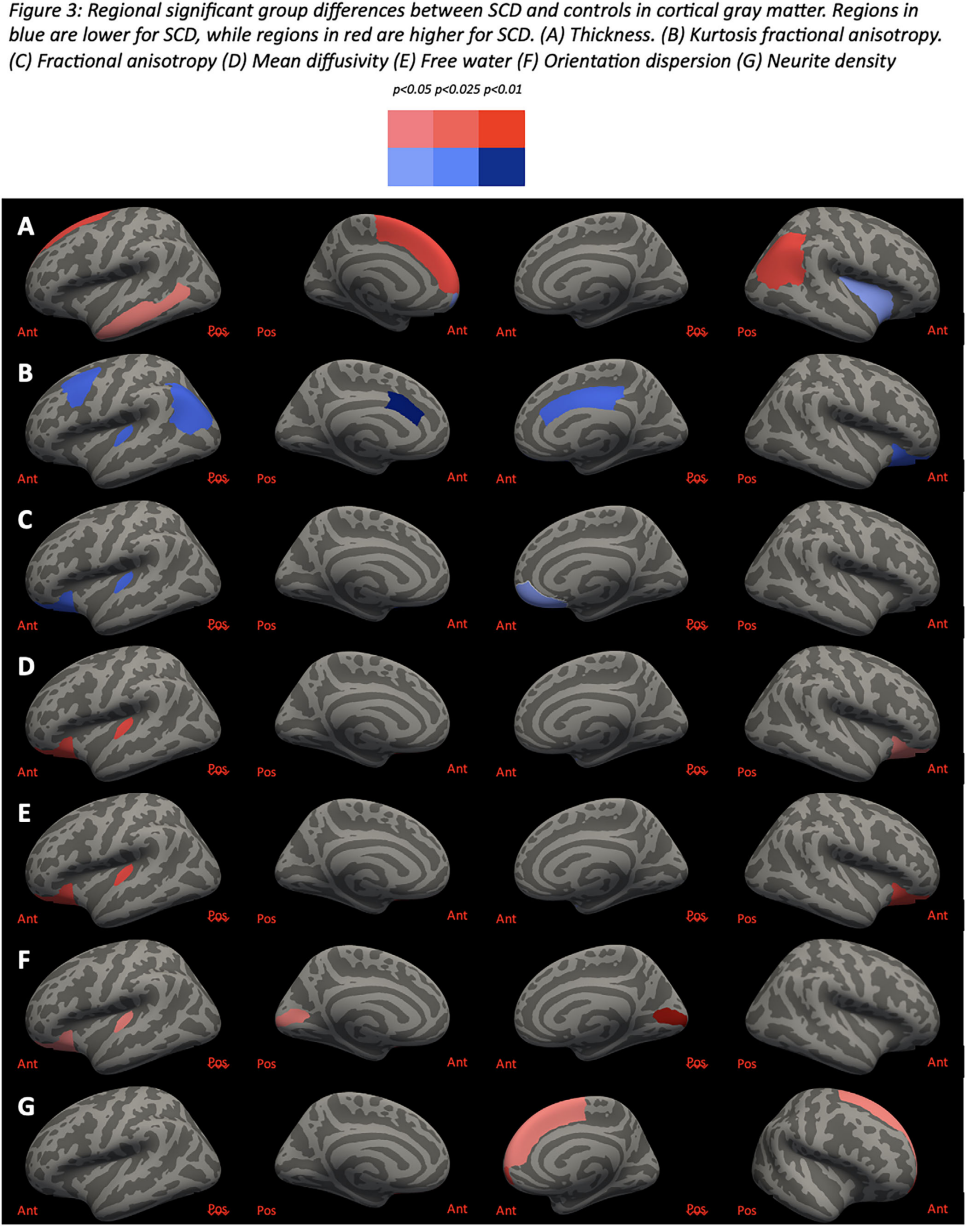# A diffusion kurtosis MRI signature of subjective cognitive decline

**DOI:** 10.1002/alz.093982

**Published:** 2025-01-09

**Authors:** Ryn Flaherty, Yu Veronica Sui, Mu Li, Ana Ardelean, Henry Rusinek, Arjun V. Masurkar, Mariana Lazar

**Affiliations:** ^1^ NYU Grossman School of Medicine, New York, NY USA; ^2^ New York University, New York, NY USA

## Abstract

**Background:**

Diffusion weighted imaging (DWI) of brain white matter is better at predicting future cognitive decline and medial temporal lobe atrophy than cerebrospinal fluid biomarkers in subjective cognitive decline (SCD)1. However, few studies have investigated gray matter DWI in SCD, despite the importance of regional gray matter changes as a biomarker for Alzheimer’s Disease and related dementias.

**Method:**

316 cognitively normal participants from Cam‐CAN2 (123 SCD) older than 55 were included in the analysis. Participants were placed in the SCD group if they endorsed problems with their memory. Imaging data was collected on a 3T Siemens Trio scanner2. Diffusion imaging included 0, 1000 and 2000 s/mm2 b‐values and 30 gradient directions, and were processed using MrTrix3, PyDesigner, and AMICO to apply diffusion tensor (DTI), diffusion kurtosis (DKI), and neurite orientation density and dispersion (NODDI) models. FreeSurfer with T1w and T2w images was used to segment subcortical and cortical regions (Figure 1B&C), then calculate regional volume and cortical thickness. Segmentations were also registered to diffusion images to generate regional averages of the diffusion metrics. Uncorrected p<0.05 according to Wilcoxon tests were considered significant.

**Result:**

The metric with the most regional group differences was kurtosis fractional anisotropy (KFA) from DKI, an indicator of structural complexity. KFA was significantly lower in SCD in 17 of 84 gray matter regions, including hippocampus (Figure 2‐3B). DTI showed reduced fractional anisotropy (Figure 2‐3C) and increased mean diffusivity (Figure 2‐3D) in SCD, but for fewer regions than KFA. NODDI showed increased free water (Figure 2‐3E), increased orientation dispersion (Figure 2‐3F), and increased neurite density (Figure 3G) in SCD. Volume and thickness were higher in SCD in 5 regions, but lower in 3 regions (Figure 2‐3A). Only KFA showed group differences in hippocampus.

**Conclusion:**

KFA was more sensitive to SCD and more consistent than regional gray matter volume, cortical thickness, and all other diffusion metrics. This may indicate KFA is a biomarker signature of SCD across the whole brain gray matter.

**References**:

• Selnes P, Aarsland D, Bjornerud A, et al. J Alzheimers Dis. 2013;33(3):723‐36.

• Shafto MA, Tyler LK, Dixon M, et al. BMC Neurol. 2014;14(1):1‐25.